# Strengthening the evidence base for nutrition and cancer in low and middle income countries

**DOI:** 10.7189/jogh.06.020306

**Published:** 2016-12

**Authors:** Isabelle Romieu, Barrie Margetts, Simón Barquera, Fabio da Silva Gomes, Marc Gunter, Nahla Hwalla, Ellen Kampman, Michael Leitzmann, Nancy Potischman, Nadia Slimani, Este Vorster, Walter C. Willett, Pattanee Winichagoon, Martin Wiseman, for the International Cancer Research Funders Nutrition Working Group

**Affiliations:** 1 Nutrition and Metabolism Section, International Agency for Research on Cancer, Lyon, France; 2 Faculty of Medicine, University of Southampton, Southampton General Hospital, Southampton, UK; 3 Centro de Investigación en Nutrición y Salud, Instituto Nacional de Salud Pública, Cuernavaca, Mexico; 4 Food, Nutrition and Cancer Division, National Cancer Institute of Brazil (INCA), Rio de Janeiro, Brazil; 5 Department of Epidemiology and Biostatistics, School of Public Health, Imperial College London, London, UK; 6 Faculty of Agricultural & Food Sciences, American University of Beirut, Beirut, Lebanon; 7 Division of Human Nutrition, Wageningen University, Wageningen, the Netherlands; 8 Department of Epidemiology and Preventive Medicine, University of Regensburg, Regensburg, Germany; 9 Division of Cancer Control and Population Sciences, National Cancer Institute, Bethesda, MD, USA; 10 Centre of Excellence for Nutrition (CEN), Faculty of Health Sciences, North-West University, Potchefstroom, South Africa; 11 Department of Nutrition, Harvard School of Public Health, Boston, MA, USA; 12 Institute of Nutrition, Mahidol University Salaya, Nakhon Pathom, Thailand; 13 World Cancer Research Fund International, London, UK

The rapid changes in nutritional patterns and ways and conditions of life occurring in in low– and middle–income countries (LMICs) are likely to be adversely affecting the incidence and survival rates of most cancers. These rapid changes raise challenges and opportunities in research on nutrition and cancer worldwide, and highlight the need for an integrated approach to determine global strategies to better understand, prevent and control the impact these changes are having on the cancer epidemic.

Although awareness of the importance of cancer in LMICs is increasing, there is still the misperception that infectious diseases represent the primary health issue in LMICs, and that cancer risk is not preventable or modifiable, and is not strongly related to nutrition. Historically, infection–related cancers (ie, cancers of the liver, stomach, and cervix) were more common in LMICs. However, the rapid expansion of modern ways of living, dietary patterns and food production observed in LMICs, particularly among the poorest people and communities, is leading to changes in disease patterns and cancer types. Currently, the most frequently diagnosed cancers in LMICs are tumors of the lung, prostate, female breast, stomach, liver, colorectum, cervix, and oesophagus [1]. Taking a global perspective, the number of incident cancers in LMICs reached eight million in 2012 and is expected to continue to rise [1]. While the increase in cancer burden may be partly explained by demographic changes, altered ways of living and dietary factors related to globalization (eg, increased consumption of highly processed foods, red meat and sugar–sweetened beverages, and increasing sedentary behaviour) are also being increasingly recognized as major contributors to the increase in cancer burden [2].

Traditional diets in LMICs have differed in their quality and quantity, but over the past few decades dietary patterns have been changing rapidly and converging towards a diet high in energy, saturated and *trans* fat, added sugar, processed food products, and generally low in micronutrient–dense foods such as fruits, vegetables, legumes (pulses) and whole grains [2]. While the cancer burden attributable to obesity is still larger in HICs than LMICs, the increasing prevalence of overweight and obesity appears to have a substantial contribution to cancer burden in Latin America, the Middle East, North Africa and parts of Asia [3]. The economic impact of overweight and obesity (US$ 2 trillion per annum) is now as great globally as that of tobacco, according to a recent analysis from the McKinsey Global Institute [4], affecting all stratum of the societies worldwide, including the most vulnerable population groups (eg, children, low socio–economic groups) contributing to greater social inequalities.

To date, most evidence linking nutrition to cancer comes from HICs, where the combination of risk factors and exposures may differ from those in LMICs. Many LMICs now face a double burden where rates of overweight and obesity and related non–communicable diseases (NCDs) are increasing, while undernutrition persists. A major challenge is to capture and to better understand the interplay between early–life and current dietary exposures that can alter infants’ and children’s growth patterns, metabolism, risk of obesity and chronic diseases in adulthood.

While the double–burden of malnutrition persists in many LMICs, changes in food systems and the wider socio–ecological determinants of these changes currently unfolding in LMICs, adds to the increased risk of NCDs, but also provides an important window of opportunity to study the impact of these changes on risk of NCDs and cancer in particular. Understanding how local, national, and international food systems shape consumption is important to help guide local policy responses. Food consumption and dietary choices are culturally and economically structured and while food is merely seen in some cultures as a source of energy for the body, other cultures consider it an element of social bonding and an essential feature of their cultural or religious experiences. To influence positive changes and to protect desirable culinary traditions, it is vital that the link between culture and nutritional choices be acknowledged, understood and addressed for each specific context.

Current UN and international recommendations linked to the prevention and control of NCDs do not adequately reflect the dynamic changes and complexity of exposure in LMICs and may not be appropriate for LMICs. Although urgent action is required, interventions need to be evidence–based and evaluated to ensure that the most critical causes are being addressed.

Key to assessing the impact of current and future nutritional exposures is the capacity to measure these exposures in an appropriate way. This capacity is lacking in many LMICs. Both for research and surveillance LMICs need to strengthen their capacity to collect suitable measures of nutrition–related behaviours. The methods need to provide sufficient detail to allow the determination of food and dietary components and contaminants, to capture their variability and change over time, and to address new and important questions that will arise in the future. The tools and methods employed need to be adaptable to the local circumstances in which they are used while retaining core qualities. Ideally, methods and exposure (and endpoint) measurements should be standardised across countries to enable cross–country comparability and pooling of results so that changing patterns of behaviour can be distinguished from methodological variability and interventions can be appropriately designed by taking into consideration educational levels and culturally sensitive practices of different populations. There is a need to establish where and how biological markers of these exposures can be used or developed, where and how the underlying biological mechanisms can be studied and what the technical requirements are to assure quality in these methods. These are not easy questions to answer, however there is much experience from centres in HICs (such as in Europe, the US, and Canada), without making assumptions and over–generalising, that can be used to help develop the protocols, train and support staff, and develop the tools and methods required for LMICs. In addition necessary financial resources must be assigned to support research needs in LMICs.

Photo: Vegetables, fruit and chicken for sale in a market in Ogbomoso, South-Western Nigeria. Courtesy of Davies Adeloye, personal collection.

Population surveys, etiologic and intervention studies, as well as implementation research are all important in developing the evidence base to tackle the rise in cancers associated with changing dietary, and physical activity patterns and food production and availability. Repeated transversal evaluation in representative samples will enable the capture of baseline information as well as changes over time at the population level. Prospective epidemiologic cohorts have solidly established their scientific value for evaluating exogenous and endogenous exposures in relation to cancer, with primary advantages being the ability to measure exposures before the onset of disease and to evaluate numerous disease outcomes within a single study. Of the more than 50 epidemiologic cancer cohorts in the US National Cancer Institute’s Cohort Consortium, only a handful are from LMICs. Cohort studies conducted in LMICs would be a valuable resource ideally positioned for novel contributions to the understanding of cancer aetiology and survival. Some longitudinal studies have already been initiated in LMICs (such as the ones included in the Consortium of Health–Orientated Research in Transitioning Societies – COHORT [5]), and building on these initiatives may prove very informative and cost–efficient.

With technological advances and the above–mentioned matters being resolved, modern techniques (metabolomics, proteomics, transcriptomics, genome–wide association study [GWAS] and epigenome–wide association study [EWAS]) offer exciting opportunities to enhance our understanding of the dysregulation of cellular metabolism in cancer and the roles of dietary, lifestyle and environmental exposures in modulating cancer processes. Their large–scale application at a population level requires technological (eg, stable high throughput methods, bioinformatics), logistical (eg, appropriate biological samples), and statistical (eg, sufficient study power) resources. In order to facilitate the translation of such methodologies to population studies, priority should be given to the development of standardized technology for appropriate collection and long–term storage of biological samples, particularly blood and DNA, tumor specimens, associated normal tissue, urine and hair. In addition, strategies for implementing the collection, in existing or new cohorts, of stool, saliva, and other relevant biological samples that could allow studying the impact of diet on the microbiome and the latter’s role in human metabolism and disease promotion and prevention should be given serious consideration. Staff training is a vital component in the expansion and development of the use of these new technologies around the world.

We have highlighted what is required to better understand the problems and challenges facing LMICs, as well as identified some of the key requirements for solving these problems. Together with the implementation of major public health control programs (eg, tobacco control, limited consumption of sugary drinks, regulation of pesticides use), a high priority is to build capacity in LMICs to undertake high quality research and to provide high quality information to support government policy and action plans. This increased capacity is for better trained staff as well as the provision of suitable infrastructure and technical support. LMICs need the capacity to set their own research priorities and agenda based on their local needs. Building capacity requires a long–term investment, but there are short– and medium–term actions that can build capacity in an incremental way. Different research institutions and UN agencies need to coordinate and harmonize their actions to maximize the impact. A critical step is to have a better understanding of what is already in place, and where the gaps and opportunities are to begin to move forward.
